# Informal dementia care: The carer’s lived experience at the divides between policy and practice

**DOI:** 10.1177/14713012221112234

**Published:** 2022-07-15

**Authors:** Anthony Britton, Martina Zimmermann

**Affiliations:** Founder Trustee, The Pam Britton Trust for Dementia, Leamington Spa, UK; UKRI Future Leaders Fellow and Lecturer, Health Humanities and Health Sciences, Department of English, 4616 King’s College London, London, UK

**Keywords:** Alzheimer’s disease, dementia, Department of Health and Social Care, health care, local authorities, policy, spouse

## Abstract

Support for informal dementia care at a local community level is not working for most carers today. Carers looking after a person with dementia have long lamented the absence of an empowered named support and an effectively actioned care plan. Drawing on literary writing and social research, we argue in this article that these challenges have existed since dementia emerged as a major condition in the West during the 1980s. Based on this historical context, we ask: Why has this issue persisted over the last four decades? How have healthcare politics and policy initiatives responded to these requests? And what can we learn from this for the current, COVID-19 exacerbated crisis of care? This article focuses on the English context, to discuss these ongoing challenges in the light of a series of policy papers, and to ask what is hampering the implementation of such policy initiatives. In England, local authorities are responsible for dementia support. This article focuses on the situation in a county in the Midlands where one of us (AB) has been lobbying local government for over a decade. The discussion contextualises the lived experience of dementia care within the situation exacerbated by the COVID-19 pandemic, ensuing politics of crises and persistent emphasis on cure over care. We find that the absence on two points centrally challenges care: a joined-up approach between health and social care and adequate information on available care support services, accessible through an empowered named contact. To enhance the lived experience of dementia care, consistent provision of individual named support and professional care support, as and when required, should become essential to local implementation of the care policy.

## Introduction

The sorrow, pain and loneliness of the informal carer of a person living with Alzheimer’s or any other type of dementia has been a theme in literary writing and other cultural productions, since the condition became a major public health concern during the 1980s (Fox, 1989). It has also been central to social dementia studies and keeps surfacing in policy efforts. Yet, as the COVID-19 pandemic has brought into sharp relief, the lived experience of dementia care continues to be dominated by feelings of isolation, exhaustion and perceived lack of support. In this article, we reflect on the lived experience of one of us, a carer and activist at the coalface, against the background of four decades of dementia care as expressed in life-writing, traced in social studies and still insufficiently addressed by policy efforts. Its aim is to illustrate how strongly the lived experience of dementia care is shaped by the presence (or not) of two things: a joined-up approach between health and social care and adequate information on available support services, accessible through an empowered named contact. Our deliberations centre on the UK context, and England in particular, but similar concerns have surfaced across other European nations, if not most prosperous countries of the Global North.

## Forty years of dementia care in literary writing and social research

By the time dementia, through attention especially to Alzheimer’s disease, had become a major public concern during the 1980s, there was little support available to informal carers. Carers could rely on some advice literature that explained the condition’s impact on the behaviour and capabilities of the person living with dementia (Cohen & Eisdorfer, 1986; Mace & Rabins, 1981), but such guides could do little to provide a sense of community. Ensuing carer activism eventually contributed to developing the relevant infrastructure including Alzheimer’s societies in many countries (Goldman, 2017: pp. 193–215). As part of this activism, a growing body of dementia life-writing, including memoirs and diaries by carers, appeared from 1988, which in itself fulfilled the purpose of advice literature (e.g. Spohr, 1995; Wall, 1996; for analyses, see, e.g. Falcus & Sako, 2019: pp. 42–79; Zimmermann, 2017: pp. 23–73). Raising awareness of the challenges of dementia care, such texts have the potential to contribute to improving healthcare (Bitenc, 2020: p. 183; Zimmermann, 2020).

The study of carer life-writing from the 1980s onwards reveals that, at least from the perception of the informal carer, nothing much has changed in terms of the felt absence of support (e.g. Gillies, 2009; Magnusson, 2014). This is corroborated by sociological research that identifies structural factors impacting on the caring experience. A review of studies between 2007 and 2019 finds that carers’ unmet needs fall into four categories. The carer requires (1) emotional support, (2) support in managing being a carer, for example, by adapting to the ever changing situation, (3) support in providing care, including access to services, and (4) support in the form of knowledge of dementia. Many interventions during this period stress that, irrespective of different situations and care trajectories, carers express the same needs, the biggest challenge being constantly necessary adjustment to the ever changing disease progression. At such breaking points in care progression, carers often do not know whom to contact and how ‘to “navigate the system”’ (Clemmensen et al., 2020: pp. 4–8, 9; see also Bieber, Nguyen, Meyer et al., 2019; Gibson et al., 2019; Heinrich, Uribe, Roes et al., 2016; Jennings et al., 2018; Stephan et al., 2018). This resonates with evidence that accessibility to services is frequently hampered by not knowing about these at the right time (Böckberg et al., 2015: p. 413).

To overcome such issues, informal carers demand ‘personally focused care’, where all the relevant support is accessible through a ‘trained link worker’ (Magnusson, 2014: pp. 293, 297–298). A care plan and a named support, or ‘trained link worker’, are exactly what carers keep emphasising in carer support group meetings, organised by one of us (AB), after he had cared for his wife with dementia from 2008 until her early death in 2013. One carer asserted that their non-dementia medical condition had been managed by a care plan so that they could feel looked after; in the care for their spouse they feel left alone and without control. Another carer lamented that they were overwhelmed by leaflets displaying myriad telephone contacts; but they had to call many different people to find out how to handle a specific situation. A contactable caring companion with continuity of involvement, trained and experienced in dementia care, properly empowered and resourced, and with a manageable caseload would make it so much easier.

According to the 2014 Care Act, people who have been allocated local authority support (as is the case for dementia) should get a care plan, including general practitioners (GPs) providing annual checkups for patients, and carers are entitled to an assessment. Also, the call for a named person is not new, but the evidence base for dementia case management is still equivocal in the UK. There have been a number of attempts to have such care managers in several parts of social care and the National Health Service (Dening et al., 2011; Piercy et al., 2018), and current research seeks to gather data that would warrant a significant shift in spend (Frost et al., 2020). Still, life-writing, sociological research and our own anecdotal evidence all highlight that the lived experience of dementia care entails lack of information and is marked by fragmented, if not inaccessible services, which could be addressed by a named contact with continuity of care (e.g. Tuijt et al., 2021). This brings us to the question as to how policy initiatives responded to these requests.

## The policy response: Cure eclipses care in person-centred strategies

Policy-focused awareness campaigns were initially driven by the National Institute on Aging (NIA; created as an agency of the US Department of Health, Education and Welfare in 1974), whose influence spreads well beyond the USA. The NIA claimed to balance the need for cure with support for those in caring relationships, but from its inception advocacy to find a cure outshone the need for carer relief (Ballenger, 2006: p. 115). The effects of this imbalance are palpable to the present day.

Recommendations for best practices in dementia healthcare exist (Fillit et al., 2006), but to date there is only symptomatic treatment available, anticholinesterases like donepezil having been in use since the 1990s. Long-term clinical trials, however, did not find these drugs cost effective (Courtney et al., 2004), inducing the National Institute for Health and Care Excellence no longer to recommend their use in mild and moderate cases of dementia. Carer advocacy eventually overturned this decision in 2010, but anticholinesterase treatment remains symptom-modifying and its effect of short duration. Concurrently, since the 1990s, there has been great expense on drug development targeting amyloid deposits in the brain. The validity of this approach has been challenged for years and further contested by a series of failed clinical trials (e.g. Herrup, 2015; Panza et al., 2014; Smith, 2021). In 2021, one such amyloid-targeting compound, aducanumab, has been approved by the Food and Drug Administration, but its disease-modifying capacity is doubted by many, not only because approval was based on limited evidence (Cross, 2021), further highlighting the pressure to find a cure (Zimmermann, 2020: pp. 135–142). With an emphasis on cure comes the insistence on early diagnosis, but as Brayne and Kelly (2019) observe, a drive for early diagnosis requires adequate post-diagnostic treatment, support and services.

Overall, policy-driven efforts to find a cure have eclipsed attention to care (Baker, 2011). Work during the 1990s called for a greater emphasis on caring medicine in old age (Callahan, 1992), while concurrent anthropological studies confirmed the continued neglect of the challenges of care (Davies, 2017; for a global perspective, see Woodward, 2012). Yet, cure keeps dominating policy initiatives. *The Prime Minister’s Challenge on Dementia* calls for ‘high-quality relationship-based care and support for people with dementia’, but only a small fraction of funding was allocated to improving care (Department of Health, 2012: pp. 6–7). And similarly, *Living Well with Dementia* (Department of Health, 2009) favours a clinic-based medical model rather than a disability model of support for people with dementia (Iliffe, 2010: p. 372). To tackle this continued imbalance, the case has meanwhile been made for withdrawing symptomatic treatment from state-funded healthcare to encourage ‘a more person centred approach […] and increased scrutiny of policy makers and commissioners to ensure adequate support for patients and their caregivers’ (Safon & Suhard, 2018: p. 64; Walsh et al., 2019: p. 2).

That said, policy documents recognising the need to hear the voice of service users have been published since the early 2000s (Table 1). These documents essentially admit that informal care has been insufficiently recognised (Fraser, 2017: p. 23). As one of the first policy papers, *Putting People First* projected the ‘creation of a truly personalised care system’ (Department of Health, 2007: p. 3). It had a powerful vision and promised the necessary funding, accepting that:

real change will only be achieved through the participation of users and carers at every stage. It recognises that sustainable and meaningful change depends significantly on our capacity to empower people who use services and to win the hearts and minds of all stakeholders […] Local government will need to spend some existing resources differently and the Government will provide specific funding to support system-wide transformation. (Department of Health, 2007: p. 2)

**Table 1.** Recent national policy documents related to dementia care.

Year	Policy document	Attention to carer
2007	Putting people first: A shared vision and commitment to the transformation of adult social care	User-centred care system
2009	Living well with dementia: A national dementia strategy	Pathway of care, agreed plan, dementia advisor
2012	Prime Minister’s challenge on dementia: Delivering major improvements in dementia care and research by 2015	Relationship-based care
2015	Prime Minister’s dementia challenge 2020	Named contact
2018	After a diagnosis of dementia: What to expect from health and care services	Care plan, named coordinator

In the wake of *Putting People First*, *Living Well with Dementia* was an important step towards recognising the need for more concrete carer support. The document specifies that, at diagnosis, the person with dementia and their carer would receive the contact details of a ‘dementia adviser’, who would provide ‘a single identifiable point of contact with knowledge of and direct access to the whole range of local services available’ (Department of Health, 2009: p. 40). But indicative of the slow implementation of such change, the most recent policy document, *After a Diagnosis of Dementia*, published by the Department of Health and Social Care (DHSC) in May 2018, braves the same issues.

Such iterative emphasis suggests that change remains incremental (Cavendish, 2019; Dixon, 2020; Neuberger, 2008). True, for innovation to happen, the meaning of this innovation has to be the same for those effecting and those adopting the change (Greenhalgh et al., 2004; Koch & Iliffe, 2011: p. 494). In addition, as Koch and Iliffe have pointed out, commissioners need evidence of effectiveness to be able to justify investment in new resources (2011: p. 488). This brings us to the local level of administration, where social care is commissioned and allocated.

## National funding versus local implementation: COVID-19 as spotlight

Dementia care is within the remit of Adult Social Care and Health. In England, this is managed locally, not nationally as part of the National Health Service. This means that healthcare provision and social care support are not only provided by separate entities; they are also funded in different ways. As our example we take the situation in a county in the Midlands. Here, at the time of writing in February 2022, *After a Diagnosis of Dementia* guidance has yet to be implemented.

In his capacity as support network initiator, AB has witnessed firsthand that the current COVID-19-related care crisis is only the most recent evidence for how poorly supported people with dementia and their informal carers continue to be locally. The Care Quality Commission (CQC), the independent regulator of health and social care in England, has observed that the pandemic ‘is shining a light on existing inequality in the health and social care system’ (2020: p. 33). The estimated value of care provided since the beginning of the pandemic in the Midlands has been calculated at £20.3 billion (Carers UK, 2020: p. 5), and the Alzheimer’s Society (2020) has estimated that, between March and September 2020, i.e. the period including the pandemic’s first lockdown, 92 million hours of informal care have been provided in the UK to people with dementia in their own homes. There was significant media attention on how older people in care homes were devalued by the medical establishment and politicians (O’Neill, 2020), and there was broad press coverage regarding the ageist nature of shielding strategies (Schräge-Früh & Tracy, 2020). Yet, comparatively little attention was paid to the situation of informal carers, who were unable to receive hands-on support during and beyond the first lockdown between March and June 2020, let alone the longer and harder winter lockdown 2020/21 (but see Cahill, 2020). We find that this absence of discussion is symptomatic of the continued lack of sustained reform and revision of healthcare versus social care support. It tells the same story of perceived lack of attention that dementia life-writing and sociological interventions reveal. Some carers might be lucky to have family, friends or neighbours so that they might receive some short respite – but when the door shuts or the video link ceases, they are again on their own. The opportunity to consult accessible and personalised online resources (Dale et al., 2018) or a phone call to or leaflets from social care are only so useful – especially if the person answering the call has little or no training and only limited life experience of dementia care.

The COVID-19 pandemic was exceptional for everyone. Yet, it has been termed the ‘geriatric emergency of 2020’ (Bonanad et al., 2020). Invoking a time of crisis, however, ‘misrepresents the duration and scale of the situation’, argues the late cultural theorist Lauren Berlant; it is a ‘redefinitional tactic, an inflationary, distorting, or misdirecting gesture’ that tries to give the impression of a sudden event, because the structural challenges never previously engendered the action that would actually be required of a crisis (2011: p. 101). In other words, so-called times of crisis bring out what has not been working for a long time. During the pandemic, imposed isolation radically changed the model of care, including the impossibility to access ambulatory services and care assistance, which further exacerbated psychological impact on carers and people with dementia (Altieri & Santangelo, 2021; Cohen et al., 2020). It is our opinion that the situation of dementia carers would have been much more manageable during the pandemic, had efforts been made to reduce fragmentation of care, as formulated in policy papers a dozen years since: ensuring a joined-up approach between health and social care and adequate information on available care support services, accessible through an empowered named contact. The question remains as to why local implementation is not forthcoming.

## Our analysis and prescription for change

Based on our experience in lobbying local authorities, we believe the main reasons are two: allocation of resources and lack of clarity, in the details of policy documents, as to who carries the responsibility as named contact. The first policy documents had recognised that a ‘dementia adviser’ would have to ‘work with both social care and health care services’ and would, thus, have to be jointly commissioned by local authorities and primary care trusts (Department of Health, 2009: pp. 40–41). Confirming the challenge of this prerequisite, reactions to the document had stressed that better services would require ‘substantial redirection of resources’ (Greaves & Jolley, 2010), explained that only hard-won evidence could induce local commissioners to invest in different services (Koch & Iliffe, 2011: p. 488), and emphasised that building a National Care Service would require that conditions like dementia be seen as disabilities requiring care rather than conditions for which cures are available (Manthorpe, 2010).

Governmental position papers pledge a greater focus on care, but it remains the territory of individual local authorities to allocate resources according to needs established. But faced with continued, nationally imposed cuts to their budgets (Bottery & Babalola, 2020), local authorities may invest in easy to subcontract-out ways, for example to national charities, to ensure a certain level of support. Concurrently, we notice an increase in demand on local support charities and voluntary group leaders, who are asked to act like a named contact, albeit without the necessary resources and healthcare training. For example, the draft of *Coventry and Warwickshire’s Living Well with Dementia Strategy 2022–2027* still only promises to ‘work towards ensuring that everyone with a dementia diagnosis has a Care Coordinator and that this role is well understood’ (2021: p. 8).

Our second main concern relates to policy documents like *After a Diagnosis of Dementia* as such. We observe that it lacks specifics about who should be the named contact: Those responsible for developing a care plan can be the memory assessment service, the local council or the GP. Ambiguity about this responsibility is not unproblematic, not least since GPs report uncertainty regarding a dementia diagnosis (Koch & Iliffe, 2011: p. 490), while large-scale projects have identified that primary care, in conjunction with memory clinic support, did not have additional effects on clinical outcomes or cost (Frost et al., 2020). Our ongoing exchange with commissioners and County Council representatives suggests that the argument will continue to be circular. Commissioners are in an unenviable position. Faced with budget constraints, they have to make difficult choices. Perhaps it is time to acknowledge that, as Koch and Iliffe write, commissioning is exceptionally challenging, and commissioners ‘may lack the skills necessary in order to decide upon the most favourable change of practice’ (2011: p. 495).

Fragmentation of care is one of the core issues hampering positive carer experience. Concentrating on a network and support strategy that matches the actual situation at a local level would enable truly purposeful support (Care Quality Commission, 2020: pp. 66–67). There have been suggestions of co-operation between Health Trusts and County Social Service Teams, but we find that only a few positive outcomes have been reached that suggest willingness to explore joined-up thinking, which is known to benefit patient care (Care Quality Commission, 2020: pp. 21, 66). Local charities would greatly welcome a planned joined-up top-down bottom-up commitment to a new and appropriate unified service.

At a macro-level, of course, a deep culture shift will be needed, one that sees the need for care as much as cure and consequently would create an almost ideological commitment to share finances more evenly between transformation of care and research into cure. In the meantime, investment in a sufficiently large number of two kinds of support professionals would make an enormous difference: (1) named contacts, allocated and introduced to the person with dementia and their carers at diagnosis, who have the role of dementia advisers, regularly (e.g. monthly) check in with the carer and are able to point them to necessary resources; and (2) qualified professionals, for example Admiral Nurses, who are properly empowered and resourced, exchange knowledge with the dementia advisers and work on a case basis as and when required (for a comparable model, see Piercy et al., 2018). A care plan, delivered by the GP, would include contact details for the dementia adviser and qualified professional, stressing the continuous availability of this support network for the duration of care.

## Conclusion

We have presented our perspective of the situation in a non-metropolitan shire county. As such, our observations have no direct claim on other local authorities. But case study approaches are able to help understand complex situations (Bowling, 2014: p. 85). The recent CQC report suggests that our story is representative of communities throughout England where ‘ineffective coordination of services [is] leading to fragmented care’ (Care Quality Commission, 2020: p. 16). Furthermore, recent pan-European studies that include the UK reveal fragmentation of care as one of the central issues in effective care support (Stephan et al., 2018; Bieber et al., 2019). The measures proposed in this article would send a signal that people with dementia and their carers are fully appreciated by, and integrated in the local community. As long as care is fragmented (Robinson et al., 2010), and carers cannot swiftly access specific specialist services, when a new care situation arises, the lived experience of dementia care will continue to be exhausting, frustrating and lonely.
